# Comparative Effectiveness of Nonoperative Treatments for Acute Anterior Epistaxis in Emergency and Otolaryngology Practice: A Systematic Review and Meta-Analysis of Randomized Controlled Trials

**DOI:** 10.7759/cureus.115154

**Published:** 2026-08-25

**Authors:** Abdulelah F Alshehri, Remas M Alonazi, Abdulrahman M Alshehri, Hadeel A Algaud, Hanin N Alabdali, Ali A Alhilal, Wajd S Alotaibi, Layan S Alahmadi, Mohammed A Ababtain, Alwaleed S Alqahtani, Hani Albrahim

**Affiliations:** 1 Clinical Research, Independent Research, Riyadh, SAU; 2 College of Medicine, Imam Mohammad Ibn Saud Islamic University, Riyadh, SAU; 3 College of Medicine, Almaarefa University, Riyadh, SAU; 4 College of Medicine, Alfaisal University, Riyadh, SAU; 5 College of Medicine, King Saud bin Abdulaziz University for Health Sciences, Riyadh, SAU; 6 Emergency, Dr. Sulaiman Al Habib Medical Group (HMG), Arrayan Hospital, Riyadh, SAU

**Keywords:** anterior epistaxis, emergency medicine, epistaxis, nasal packing, tranexamic acid

## Abstract

Acute anterior epistaxis is frequently managed with multiple nonoperative interventions, but comparative evidence regarding their effectiveness remains uncertain. This systematic review and meta-analysis evaluated the effectiveness and safety of nonoperative treatments for acute anterior epistaxis in emergency and otolaryngology settings.

PubMed/MEDLINE, Scopus, and Web of Science were searched from inception through 3 July 2026 for randomized controlled trials (RCTs) comparing nonoperative hemostatic interventions in adults with acute anterior epistaxis. Risk of bias was assessed using the Cochrane Risk of Bias 2 tool, and random-effects meta-analysis was performed when sufficient clinical comparability was present. Nine RCTs including 1,124 participants were identified, with three trials contributing to quantitative synthesis.

Topical tranexamic acid reduced rebleeding within 24 hours compared with matched vehicle controls (RR, 0.39; 95% CI, 0.17-0.89), with an absolute risk reduction of 19.1 percentage points. However, topical tranexamic acid was not associated with a significant reduction in further hemostatic intervention (RR, 0.59; 95% CI, 0.22-1.59). Other nonoperative strategies showed variable effects across heterogeneous outcomes.

Overall, topical tranexamic acid may reduce early rebleeding in acute anterior epistaxis, but evidence regarding broader clinical outcomes remains limited by the small number of comparable trials, heterogeneous protocols, and inconsistent outcome definitions. Further high-quality randomized trials with standardized outcomes are required to clarify the optimal role of these interventions.

## Introduction and background

Epistaxis is among the most common otolaryngological emergencies. It has an estimated lifetime prevalence of approximately 60% in the general population, and 80% to 90% of episodes arise from the anterior nasal cavity, most often from the anterior septum in the region of the Kiesselbach plexus, where the bleeding point is accessible to direct inspection and local treatment [1]. Posterior bleeding is less frequent, harder to localize, and more often requires specialist or operative management. In the United States, epistaxis accounts for approximately 1 in 200 emergency department attendances, with incidence rising steeply after the sixth decade [2]. Acute anterior epistaxis accounts for the majority of presentations, and initial management is commonly undertaken in emergency settings before specialist involvement.

Management follows a stepwise sequence. Compression of the cartilaginous nose for 10 to 15 minutes controls many episodes, and topical vasoconstrictors with a local anesthetic are applied when bleeding persists [3]. If a bleeding point is identified, chemical or electrical cautery may be used [4]. When cautery fails or no source is visible, anterior nasal packing remains standard practice, and absorbable hemostatic matrices and powders have been introduced as alternatives intended to achieve hemostasis without sustained tamponade [5].

Although effective, nonresorbable packing can be painful during insertion and removal and may cause mucosal injury, infection, pressure necrosis, and, rarely, toxic shock syndrome [3,5]. It may also prolong emergency department care and require planned removal or admission, with inpatient management accounting for a disproportionate share of epistaxis-related costs [6]. Topical tranexamic acid, delivered by pledget or atomized application, has been investigated as a pharmacological approach to improve hemostasis and potentially reduce the need for packing [7]. Selection among these options varies substantially between clinicians and units, reflecting training and local convention as much as evidence [8].

Randomized controlled trials (RCTs) have evaluated multiple nonoperative approaches, but the evidence remains difficult to apply. Interventions differ in mechanism, method of application, and degree of mechanical tamponade; comparators range from matched vehicles and topical vasoconstrictors to conventional nasal packing; and outcome definitions vary in construct and timing, with early hemostasis assessed at 5, 10, or 15 minutes and rebleeding recorded at intervals from 24 hours to 30 days. Previous syntheses have examined cautery, packing devices, topical hemostatic agents, and tranexamic acid [4,5,7].

A network meta-analysis compared 12 noninvasive local treatments across 20 trials in broader epistaxis populations that were not restricted exclusively to acute anterior epistaxis [9]. However, these reviews differed in population scope, analytical approach, and included comparators. Clinical and methodological heterogeneity continues to limit interpretation, and additional randomized trials published after these reviews warrant updated assessment.

We therefore conducted an updated systematic review and meta-analysis of RCTs evaluating nonoperative treatments for acute anterior epistaxis in emergency department and otolaryngology settings. The objective was to synthesize direct randomized evidence from these clinical settings on initial bleeding control, rebleeding, the need for further hemostatic intervention, healthcare utilization, patient-reported outcomes, and safety.

## Review

Methods

Study Design and Reporting Standards

This systematic review and meta-analysis evaluated RCTs comparing nonoperative treatments for acute anterior epistaxis in emergency and otolaryngology practice. The review was conducted and reported according to the Preferred Reporting Items for Systematic Reviews and Meta-Analyses (PRISMA) 2020 statement [10]. The review was not prospectively registered.

Literature Search Strategy

PubMed/MEDLINE, Scopus, and Web of Science were searched from database inception through 3 July 2026. The search strategy combined database-specific subject headings and free-text terms related to acute anterior epistaxis, nonoperative hemostatic interventions, and RCTs. Search syntax was adapted for each database. The search strategy is presented in Table 1. Reference lists of eligible studies were manually screened, and duplicate records were removed before screening.

**Table 1:** Search strategy used for the systematic review.

Search component	Search terms
Condition	epistaxis OR “anterior epistaxis” OR nosebleed
Intervention	“tranexamic acid” OR “topical hemostatic agent*” OR “nasal packing” OR “nasal tampon” OR “Rapid Rhino” OR Merocel OR Floseal OR “oxidized cellulose” OR Traumastem
Study design	random* OR “randomized controlled trial” OR “randomised controlled trial”
Combined search strategy	(epistaxis OR “anterior epistaxis” OR nosebleed) AND (“tranexamic acid” OR “topical hemostatic agent*” OR “nasal packing” OR “nasal tampon” OR “Rapid Rhino” OR Merocel OR Floseal OR “oxidized cellulose” OR Traumastem) AND (random* OR “randomized controlled trial” OR “randomised controlled trial”)

Eligibility Criteria

Eligibility criteria were defined according to the PICOS framework. Studies were eligible according to predefined population, intervention, comparator, outcome, and study-design criteria. Detailed inclusion and exclusion criteria are presented in Table 2.

**Table 2:** Inclusion and exclusion criteria for study eligibility.

Eligibility criterion	Inclusion criteria	Exclusion criteria
Population	Adults with acute anterior epistaxis managed in emergency departments, ENT emergency departments, or otolaryngology services	Pediatric-only populations
Epistaxis type	Acute anterior epistaxis	Posterior, postoperative, nonacute, or nonanterior epistaxis not addressing the predefined review question
Intervention	Nonoperative hemostatic treatments, including topical tranexamic acid, hemostatic matrices or absorbable agents, nasal-packing devices, compression-based strategies, and other conservative interventions	Ineligible interventions
Comparator	Matched control or placebo, conventional anterior nasal packing, alternative nasal-packing devices, or other eligible nonoperative treatments	Ineligible comparators
Outcomes	Initial bleeding control, rebleeding, further hemostatic intervention, anterior nasal packing, emergency-department length of stay, hospital admission, re-presentation, pain, discomfort, satisfaction, or adverse events	Insufficient extractable outcome data
Study design	Randomized controlled trials	Nonrandomized studies, observational studies, case series, reviews, or protocols without outcome data
Dataset	Eligible unique study datasets	Duplicate or overlapping datasets

Study Selection and Data Extraction

Titles and abstracts were screened, followed by full-text assessment of potentially eligible reports against predefined eligibility criteria. Data were extracted using a standardized extraction form. Extracted variables included author, publication year, country, clinical setting, trial design, randomized sample size, participant demographics, intervention and comparator characteristics, outcome definitions, assessment times, follow-up duration, event counts, continuous outcome data, adverse events, and healthcare-utilization outcomes.

Risk-of-Bias Assessment

Risk of bias was assessed using the Cochrane Risk of Bias 2 (RoB 2) tool. The assessed domains were bias arising from the randomization process; bias due to deviations from intended interventions; bias due to missing outcome data; bias in measurement of the outcome; and bias in selection of the reported result. Judgments were classified as low risk, some concerns, or high risk of bias.

Because RoB 2 assessments are outcome-specific, judgments were based on outcomes contributing to the quantitative synthesis, with consideration of the primary effectiveness outcomes reported by the included trials. Risk-of-bias results were summarized using a traffic-light figure.

Data Synthesis and Statistical Analysis

Quantitative synthesis was performed only when studies demonstrated sufficient comparability regarding intervention, comparator, outcome definition, and timing of assessment. Nine RCTs involving 1,124 participants were included, while three trials contributed to the quantitative meta-analyses evaluating rebleeding within 24 hours and requirement for further hemostatic intervention. Outcomes with substantial clinical or methodological heterogeneity were synthesized narratively.

Dichotomous outcomes were pooled as risk ratios (RRs) with 95% confidence intervals using a random-effects model. The RR was considered the primary effect measure. Risk differences were calculated as secondary absolute-effect analyses, and the number needed to treat was derived from the pooled risk difference when appropriate. Findings were interpreted cautiously when relative and absolute effect measures yielded different conclusions.

Statistical heterogeneity was assessed using Cochran’s Q test, I² statistic, and τ². I² values of approximately 25%, 50%, and 75% were interpreted as low, moderate, and high heterogeneity, respectively. No publication-bias assessment or funnel plot analysis was performed because fewer than 10 studies contributed to each meta-analysis. No meta-regression or subgroup analyses were conducted because of the limited number of included studies.

Results

Study Selection

**Figure 1:**
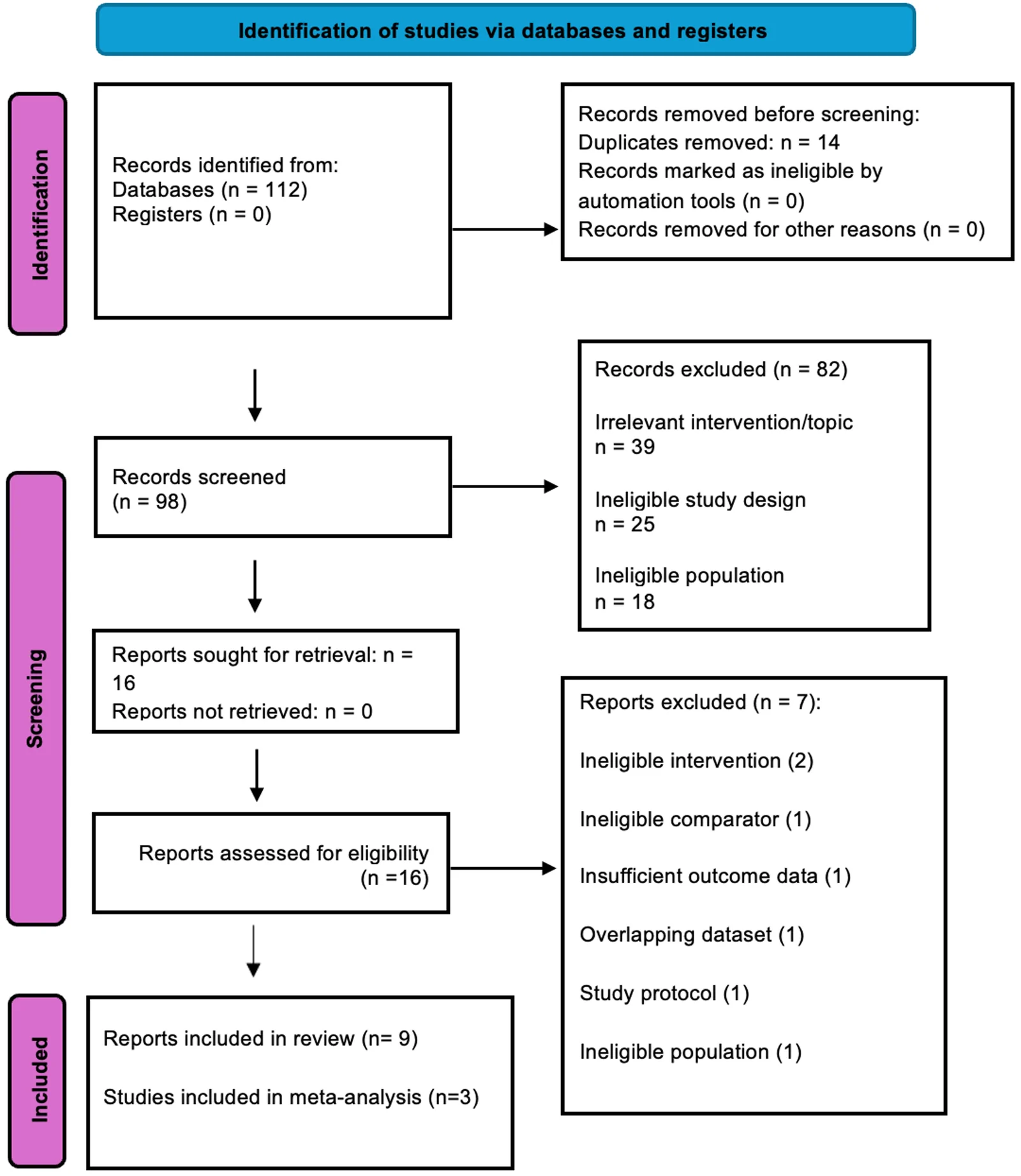
PRISMA 2020 flow diagram of the study selection process. The diagram summarizes study identification, screening, eligibility assessment, and inclusion in the qualitative and quantitative syntheses, adapted from the PRISMA 2020 statement [10]. PRISMA, Preferred Reporting Items for Systematic Reviews and Meta-Analyses

The study selection process is summarized in Figure 1. A total of 112 records were identified through database searching, with no additional records retrieved from registers. After removal of 14 duplicate records, 98 records underwent title and abstract screening. Of these, 82 records were excluded because they investigated irrelevant interventions or topics, used ineligible study designs, or enrolled populations outside the predefined eligibility criteria.

The remaining 16 full-text reports were assessed for eligibility. Following full-text review, seven reports were excluded because of ineligible interventions or comparators, insufficient outcome data, duplicate or overlapping datasets, publication as a study protocol, or failure to meet the predefined population eligibility criteria. Ultimately, nine RCTs fulfilled the eligibility criteria and were included in the qualitative synthesis. Of these, three studies contributed to the quantitative meta-analyses evaluating rebleeding within 24 hours and the requirement for further hemostatic intervention, whereas the remaining studies were synthesized narratively because of substantial clinical and methodological heterogeneity in interventions, comparator groups, outcome definitions, and follow-up duration.

Characteristics of Included Studies

Nine RCTs comprising 1,124 participants were included (Table 3). The studies were published between 2005 and 2024 and were conducted in Iran (n = 3), the United States (n = 2), Turkey (n = 2), the United Kingdom (n = 1), and Canada (n = 1). Most trials were conducted in general emergency departments (6/9), whereas the remaining studies were performed in otolaryngology services or dedicated ear, nose, and throat (ENT) emergency departments, indicating that the available evidence primarily reflects acute-care practice. Sample sizes ranged from 26 to 240 participants. Two trials were double-blind, one was partially blinded, and the remaining six were open-label. The evaluated interventions included topical tranexamic acid, Floseal hemostatic matrix, Rapid Rhino nasal packing, Traumastem powder, and alternative nasal packing strategies. Extracted outcomes included bleeding control, recurrent epistaxis, patient-reported outcomes, emergency department length of stay, and the need for additional interventions. Follow-up ranged from assessment during the emergency department visit to 30 days.

**Table 3:** Characteristics of the included randomized controlled trials. ED, emergency department; ENT, ear, nose and throat; I/C/T, intervention/comparator/total; NR, not reported; RCT, randomized controlled trial; SD, standard deviation; TXA, tranexamic acid; VAS, visual analogue scale

Study	Country	Setting	Design	Randomized (I/C/T)	Age (years)	Male (%)	Intervention	Comparator	Outcomes extracted	Follow-up
Singer et al. (2005) [11]	USA	Two emergency departments	Parallel-group, open-label RCT	20/20/40	61 (48-79), overall	67.5	Rapid Rhino nasal pack	Rhino Rocket nasal tampon	Initial hemostasis; rebleeding at removal and ≤2 days; pain of insertion and removal (100-mm VAS); ease of insertion and removal (5-point Likert)	24-72 h; ≤2 days
Mathiasen and Cruz (2005) [12]	USA	Tertiary emergency department	Parallel-group, open-label RCT	35/35/70	73.8 ± 10.8/67.8 ± 16.6	NR	Floseal hemostatic matrix with digital compression	Conventional anterior nasal packing	Initial hemostasis; rebleeding ≤7 days and at pack removal; discomfort; patient satisfaction; otolaryngology consultation	During ED stay; 5-7 days
Moumoulidis et al. (2006) [13]	UK	Tertiary otolaryngology department	Parallel-group, open-label RCT	21/21/42	71.3 (men); 72.6 (women)	54.8	Rapid Rhino 7.5-cm nasal pack	Merocel 8-cm nasal packing	Bleeding control; discomfort; additional intervention; rebleeding	24-48 h
Zahed et al. (2013) [14]	Iran	Urban teaching emergency department	Parallel-group, open-label RCT	107/109/216	50.4 ± 19.0/54.0 ± 15.5	57.4	Topical TXA 500 mg/5 mL pledget	Conventional anterior nasal packing	Bleeding arrest ≤10 min; rebleeding; satisfaction; ED discharge ≤2 h; adverse events	Discharge; 24 h; 7 days
Murray et al. (2018) [15]	Canada	Two tertiary otolaryngology services	Parallel-group, open-label RCT	13/13/26	59.0/55.1	53.8	Floseal hemostatic matrix	Conventional anterior nasal packing	Hemostasis; 30-day re-presentation; comfort; admission; adverse events	48 h; 30 days
Akkan et al. (2019) [16]	Turkey	Tertiary emergency department	Three-arm RCT (partially blinded)	45/45 + 45/135	60 (48-72), overall	NR	Atomized TXA 500 mg with external compression	Merocel nasal packing; placebo atomized saline	Bleeding cessation ≤15 min; rebleeding ≤24 h; rescue packing	15 min; 24 h
Pouraghaei et al. (2020) [17]	Iran	Two university emergency departments	Parallel-group, open-label RCT	107/96/203	51.9 ± 16.5, overall	56.2	Traumastem powder 2 g	Conventional anterior nasal packing	Bleeding control ≤5 min; rebleeding ≤24 h; satisfaction; rescue packing	Discharge; 24 h
Hosseinialhashemi et al. (2022) [18]	Iran	Academic tertiary ENT emergency department	Parallel-group, double-blind RCT	120/120/240	52 (43-61)/53 (46-63)	52.5	TXA 500 mg pledget with phenylephrine and lidocaine	Placebo pledget with phenylephrine and lidocaine	Need for anterior nasal packing; rebleeding; ED stay; cauterization; adverse events	24 h; Days 1-3; Day 7
Arikan and Akyol (2024) [19]	Turkey	Tertiary emergency department	Three-arm, double-blind RCT	51 + 50/51/152	NR	NR	TXA-soaked gauze packing (500 mg or 1000 mg)	Saline-soaked gauze packing	Bleeding control at 5 and 10 min; rebleeding; salvage therapy; otolaryngology consultation; adverse events	5 min; 10 min; 24 h

The included populations represented different stages of the clinical management pathway. Five trials enrolled patients presenting with active anterior epistaxis as first-line cases, whereas three included patients who had failed initial conservative measures or already required nasal packing. One trial focused on persistent epistaxis referred after unsuccessful previous management, representing a more refractory patient population. Overall, the included studies encompassed a broad spectrum of patients encountered in routine emergency and otolaryngology practice.

Clinical Effectiveness Outcomes

Clinical-effectiveness outcomes included initial bleeding control, rebleeding, further hemostatic intervention, healthcare utilization, and patient-reported outcomes. Studies evaluating nasal-packing devices primarily focused on device performance, hemostatic success, patient discomfort, and satisfaction, whereas trials investigating topical tranexamic acid or other hemostatic agents additionally assessed rebleeding, further hemostatic intervention, and healthcare-utilization outcomes. Emergency-department length of stay was evaluated in several emergency-department studies, whereas hospital admission and 30-day re-presentation were assessed in one otolaryngology-based study.

Initial bleeding control was assessed in eight trials using heterogeneous outcome definitions and assessment time points and was therefore not pooled. In Singer et al. [11], successful bleeding control with a single nasal tampon was achieved in 18 of 20 patients (90%) in both treatment groups (RR, 1.00; 95% CI, 0.80-1.20). In Mathiasen and Cruz [12], physician-rated effectiveness was significantly higher with Floseal than with conventional nasal packing (9.9 ± 0.4 vs. 7.7 ± 3.6; p < 0.001), and crossover because of treatment failure occurred in 1 of 35 patients (3%) receiving Floseal compared with 8 of 35 patients (23%) receiving conventional packing (p < 0.05). In Moumoulidis et al. [13], bleeding control while the nasal pack remained in situ was achieved in 17 of 21 patients (81%) treated with Merocel and 16 of 21 patients (76%) treated with Rapid Rhino (p = 0.917). In Zahed et al. [14], bleeding arrest within 10 minutes occurred in 76 of 107 patients (71.0%) receiving topical tranexamic acid compared with 34 of 109 patients (31.2%) receiving anterior nasal packing (RR, 2.28; 95% CI, 1.68-3.09; p < 0.001). In Murray et al. [15], immediate hemostasis occurred in 10 of 13 patients (76.9%) treated with Floseal and 11 of 13 patients (84.6%) treated with traditional packing, whereas hemostasis at 48 hours was achieved in 10 of 13 (76.9%) and 9 of 13 patients (69.2%), respectively (p = 1.000 for both comparisons). In Akkan et al. [16], bleeding cessation at 15 minutes occurred in 41 of 45 patients (91.1%) receiving atomized tranexamic acid with external compression, 42 of 45 (93.3%) receiving Merocel packing, and 32 of 45 (71.1%) receiving atomized saline with external compression.

The absolute differences were 20.0 percentage points (95% CI, 3.6-35.4) for tranexamic acid versus saline, 22.2 percentage points (95% CI, 6.3-37.3) for Merocel versus saline, and 2.2 percentage points (95% CI, -10.2 to 14.8) for tranexamic acid versus Merocel. In Pouraghaei et al. [17], bleeding control within five minutes was achieved in 85 of 107 patients (79.4%) treated with Traumastem (oxidized-cellulose) powder and 85 of 96 patients (88.5%) receiving routine nasal packing (p = 0.058); the median bleeding-control time was 4.0 minutes in both groups (p = 0.764). Hosseinialhashemi et al. [18] evaluated the need for anterior nasal packing as the primary endpoint rather than early bleeding cessation. In Arikan and Akyol [19], absence of bleeding at five minutes was observed in 33 of 51 patients (64.7%) receiving 1000 mg topical tranexamic acid, 26 of 50 (52.0%) receiving 500 mg, and 15 of 51 (29.4%) receiving saline (p < 0.001). At 10 minutes, bleeding had ceased in 42 of 51 patients (82.4%), 36 of 50 (72.0%), and 28 of 51 (54.9%), respectively (p = 0.010).

Healthcare-utilization outcomes were reported by five trials using non-commensurable outcome definitions and were therefore summarized narratively. In Zahed et al. [14], discharge within two hours occurred in 102 of 107 patients (95.3%) receiving topical tranexamic acid compared with 7 of 109 patients (6.4%) receiving anterior nasal packing (p < 0.001). In Mathiasen and Cruz [12], in-person otolaryngology consultation was required in 8.6% of patients treated with Floseal compared with 31.0% treated with conventional nasal packing (p < 0.05). In Murray et al. [15], hospital admission occurred in 2 of 13 patients (15.4%) treated with Floseal and 6 of 13 patients (46.2%) treated with traditional packing. Thirty-day re-presentation occurred in the same proportions (p = 0.202 for both outcomes), and the incremental cost-effectiveness ratio was -$11,891 per rebleed prevented (95% CI, -$37,658 to $473) in 2016 Canadian dollars. In Pouraghaei et al. [17], the median emergency-department stay was 85 minutes (IQR, 45-110) with Traumastem (oxidized-cellulose) powder and 60 minutes (IQR, 45-88) with routine nasal packing (p = 0.095). In Hosseinialhashemi et al. [18], an emergency-department stay exceeding two hours occurred in 11 of 120 patients (9.2%) receiving topical tranexamic acid and 25 of 120 patients (20.8%) receiving the matched vehicle (absolute difference, -11.6 percentage points; 95% CI, -20.6 to -2.8; OR, 0.38; 95% CI, 0.18-0.82).

Rebleeding within 24 hours and the requirement for further hemostatic intervention were reported with sufficient consistency of comparator and outcome definition by Akkan et al. [16], Hosseinialhashemi et al. [18], and Arikan and Akyol [19] to permit quantitative synthesis. These were the trials identified in our review that provided sufficiently comparable outcome definitions and available data for quantitative synthesis of rebleeding within 24 hours and further hemostatic intervention. Accordingly, these outcomes were considered sufficiently homogeneous for quantitative synthesis, whereas the remaining clinical and patient-reported outcomes were summarized narratively because of substantial methodological heterogeneity.

Topical Tranexamic Acid Versus Control Interventions for Rebleeding Within 24 Hours

Three RCTs comprising 482 participants (266 allocated to topical tranexamic acid and 216 to a matched vehicle) contributed to this meta-analysis. In Akkan et al. [16], only the matched-vehicle comparison was included because the Merocel nasal-packing arm did not meet the predefined comparator criterion. In Arikan and Akyol [19], the 1000-mg and 500-mg topical tranexamic acid groups were combined into a single intervention group against the shared saline control.

**Figure 2:**
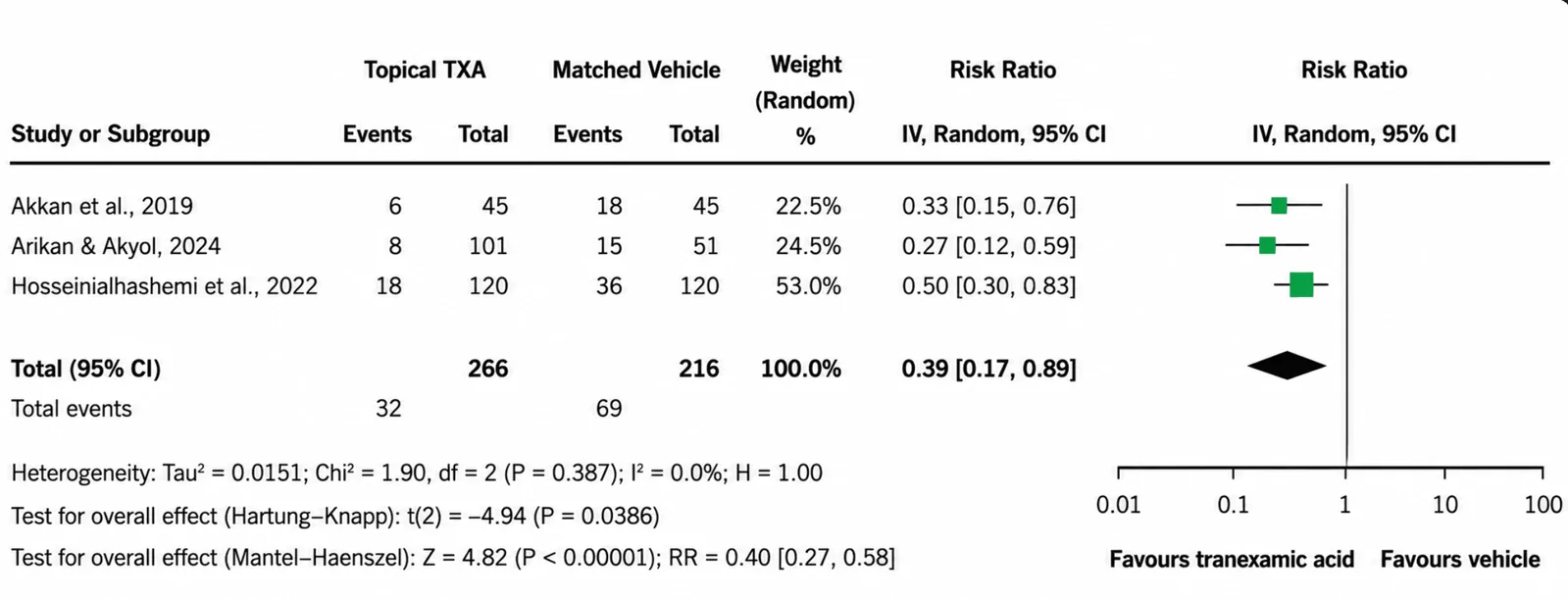
Forest plot of rebleeding within 24 hours comparing topical tranexamic acid with matched vehicle controls. The analysis included Akkan et al. [16], Hosseinialhashemi et al. [18], and Arikan and Akyol [19]. Squares represent individual study estimates, horizontal lines indicate 95% confidence intervals, and the diamond represents the pooled effect. Risk ratios below 1 favor topical tranexamic acid.

As shown in Figure 2, the pooled analysis demonstrated that topical tranexamic acid significantly reduced the risk of rebleeding within 24 hours compared with a matched vehicle (RR, 0.39; 95% CI, 0.17-0.89; p = 0.039). No statistical heterogeneity was observed (τ² = 0.0151; χ² = 1.90, df = 2; p = 0.387; I² = 0%).

The pooled control-group risk was 31.9%, and the corresponding estimated risk with topical tranexamic acid was 12.5%. On the absolute scale, topical tranexamic acid reduced rebleeding by 19.1 percentage points (RD, -0.191; 95% CI, -0.330 to -0.052), corresponding to a number needed to treat of 5 (95% CI, 3-19).

Zahed et al. [14] and Akkan et al. [16] also compared topical tranexamic acid with anterior nasal packing. That comparison was not pooled because the comparator differed from the matched vehicle and only two eligible trials contributed.

Requirement for Further Hemostatic Intervention

The same three RCTs comprising 482 participants contributed to this meta-analysis. In Akkan et al. [16], the outcome was defined as rescue treatment after failure to achieve bleeding control at 15 minutes. Hosseinialhashemi et al. [18] defined the outcome as the requirement for anterior nasal packing during ENT emergency-department attendance, whereas Arikan and Akyol [19] defined it as salvage treatment with a sponge or inflatable-balloon nasal tampon.

**Figure 3:**
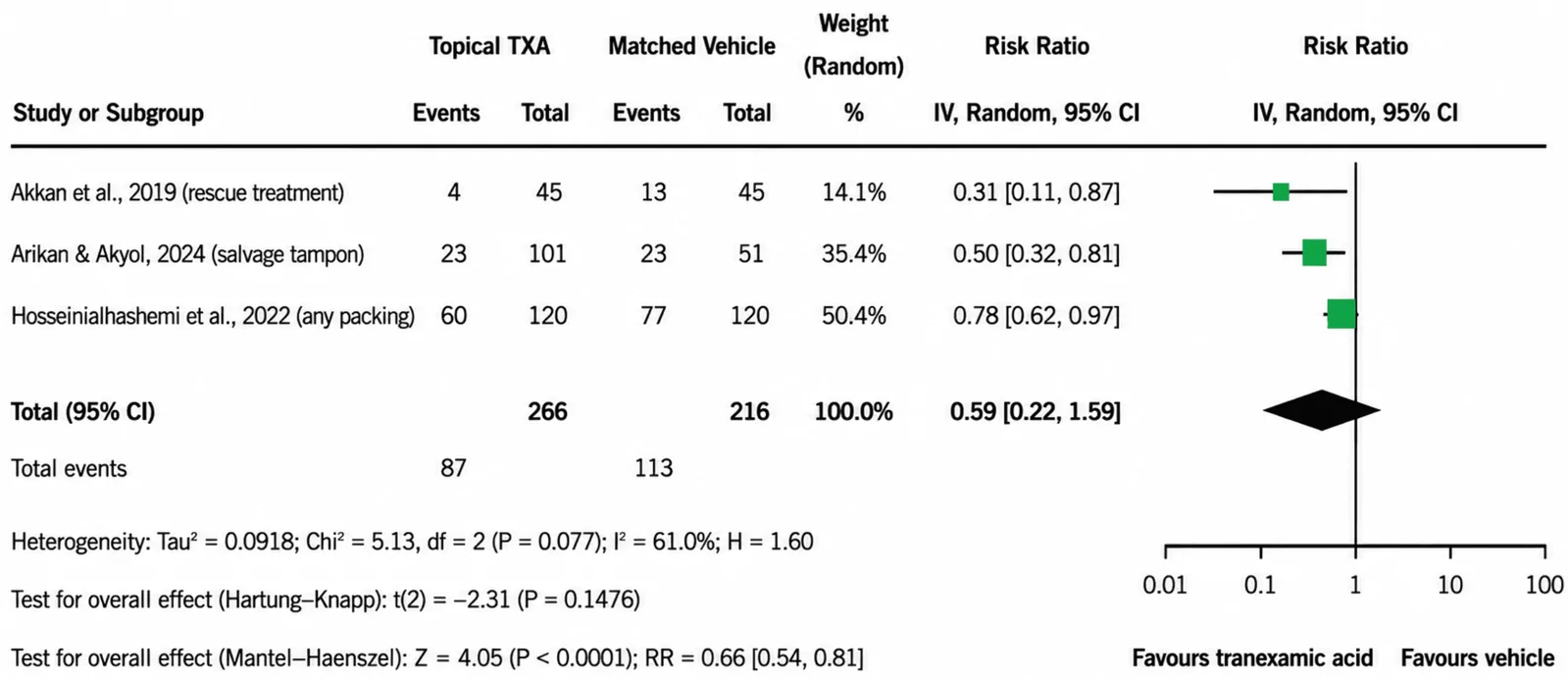
Forest plot of further hemostatic intervention comparing topical tranexamic acid with matched vehicle controls. The analysis included Akkan et al. [16], Hosseinialhashemi et al. [18], and Arikan and Akyol [19]. Squares represent individual study estimates, horizontal lines indicate 95% confidence intervals, and the diamond represents the pooled effect. Risk ratios below 1 favor topical tranexamic acid.

As shown in Figure 3, topical tranexamic acid was not associated with a statistically significant reduction in the requirement for further hemostatic intervention compared with a matched vehicle under the primary risk-ratio model (RR, 0.59; 95% CI, 0.22-1.59; p = 0.148). Moderate statistical heterogeneity was observed (τ² = 0.0918; χ² = 5.13, df = 2; p = 0.077; I² = 61%). The differing outcome definitions were reflected in the baseline event rates, with control-group risks ranging from 28.9% to 64.2%. On the absolute scale, a secondary risk-difference analysis demonstrated an absolute reduction of 18.0 percentage points (RD, -0.180; 95% CI, -0.289 to -0.072), with no statistical heterogeneity (I² = 0%).

Because the secondary risk-difference analysis yielded a different inferential conclusion from the primary risk-ratio analysis, these findings should be interpreted cautiously in the context of heterogeneous outcome definitions and substantial variation in baseline risk across studies.

Patient-Reported and Safety Outcomes

Patient-reported outcomes were evaluated in six trials but could not be pooled because the studies used different instruments, assessment phases, and reporting formats. Across studies, patient-reported outcomes consistently favored the investigational intervention, although the assessment methods varied substantially.

In Singer et al. [11], Rapid Rhino was associated with less pain during insertion than Rhino Rocket (30 vs. 48 mm on a 100-mm visual analogue scale (VAS); mean difference, 18 mm; 95% CI, 1-35). Removal pain was also lower with Rapid Rhino (11 vs. 23 mm), although the between-group difference was not statistically significant (mean difference, 12 mm; 95% CI, -1 to 25). In Mathiasen and Cruz [12], Floseal was associated with lower patient discomfort than physician-selected nasal packing at initial treatment (1.4 ± 0.6 vs. 8.9 ± 1.0) and follow-up (0.0 vs. 8.5 ± 1.2). Patient satisfaction was also higher with Floseal (9.1 ± 1.2 vs. 2.9 ± 1.6; all p < 0.0001). In Moumoulidis et al. [13], Rapid Rhino was associated with lower discomfort than Merocel during insertion and removal (both p < 0.001), whereas discomfort while the pack remained in situ was similar between groups (p = 0.979). In Murray et al. [15], Floseal was associated with lower pain during placement (2.4 vs. 7.8; p = 0.0022), treatment (0.5 vs. 4.5; p = 0.0007), and removal (0.0 vs. 3.9; p = 0.0021). Zahed et al. [14] reported greater patient satisfaction with topical tranexamic acid than with anterior nasal packing (8.5 ± 1.7 vs. 4.4 ± 1.8; p < 0.001). Similarly, Pouraghaei et al. [17] reported higher median satisfaction with Traumastem than with routine nasal packing (8.0 (IQR, 8.0-9.0) vs. 6.0 (IQR, 6.0-7.0); p < 0.001), despite no significant difference in bleeding-control time.

Safety outcomes were reported inconsistently and were therefore summarized narratively. Treatment-related adverse effects did not differ significantly among the 1000-mg tranexamic acid, 500-mg tranexamic acid, and saline groups in Arikan and Akyol [19] (6.0%, 4.0%, and 3.9%, respectively; p = 0.854). Hosseinialhashemi et al. [18] reported no minor or major adverse events after structured assessment for symptoms suggestive of deep-vein thrombosis, pulmonary embolism, or cerebrovascular events. Zahed et al. [14] found no significant difference in complications, defined as nausea, vomiting, and intolerance, between topical tranexamic acid and anterior nasal packing (4.7% vs. 11.0%; OR, 0.42; 95% CI, 0.16-1.16; p = 0.128). No treatment-related complications were reported in Mathiasen and Cruz [12], Moumoulidis et al. [13], or Murray et al. [15], whereas Pouraghaei et al. [17] reported transient sneezing in five patients immediately after application of oxidized-cellulose powder, prompting a change in treatment.

Overall, no serious treatment-related adverse events were reported among studies that assessed safety. However, safety data remain limited because follow-up in the topical tranexamic acid trials did not exceed seven days, Singer et al. [11] did not report adverse-event outcomes and acknowledged that the trial was not powered to detect small differences in adverse events, and Akkan et al. [16] did not prespecify a safety outcome. Minor adverse events were uncommon but were reported using study-specific definitions, limiting direct comparisons across interventions.

Risk-of-Bias Assessment

**Figure 4:**
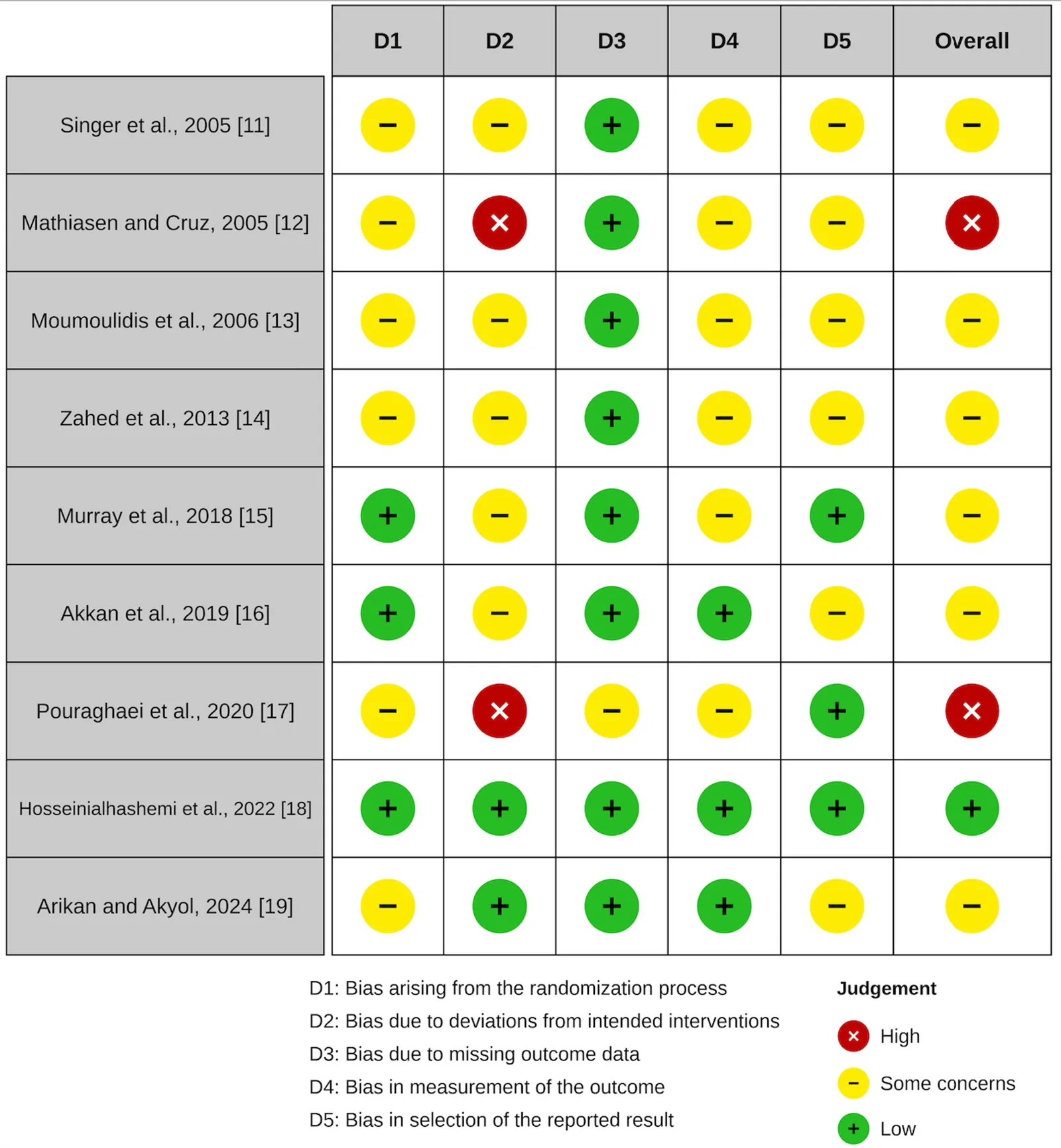
Risk-of-bias assessment of the included randomized controlled trials using the Cochrane Risk of Bias 2 tool. D1, bias arising from the randomization process; D2, bias due to deviations from intended interventions; D3, bias due to missing outcome data; D4, bias in measurement of the outcome; D5, bias in selection of the reported result. Green indicates low risk of bias, yellow indicates some concerns, and red indicates high risk of bias.

Risk of bias was assessed using the Cochrane RoB 2 tool and is presented in Figure 4. Overall, the methodological quality of the included trials was moderate. One trial was judged to be at low risk of bias, six raised some concerns, and two were judged to be at high risk of bias. The most frequent methodological limitations were insufficient reporting of the randomization process, open-label study designs that may have introduced bias due to deviations from intended interventions or outcome measurement, and limited information regarding the selection of reported results. High-risk judgments were confined to two studies and were primarily attributable to important concerns regarding deviations from intended interventions and post-randomization data handling. Notably, none of the studies contributing to the quantitative meta-analyses was judged to be at high risk of bias, supporting the overall credibility of the pooled effect estimates despite the methodological limitations observed across the broader evidence base.

Discussion

Nine RCTs involving 1,124 patients met the eligibility criteria, and three of these, comprising 482 patients, contributed to quantitative synthesis. Topical tranexamic acid reduced rebleeding within 24 hours relative to a matched vehicle (RR 0.39, 95% CI 0.17 to 0.89), corresponding to an absolute reduction of 19.1 percentage points and a number needed to treat of 5. The requirement for further hemostatic intervention was not significantly reduced under the primary RR model (RR 0.59, 95% CI 0.22 to 1.59), although the secondary risk difference analysis suggested an absolute reduction of 18.0 percentage points. The remaining six trials, which evaluated hemostatic matrices and nasal packing devices, were synthesized narratively because their interventions, comparators, and outcome definitions were not sufficiently comparable for quantitative pooling.

The reduction in early rebleeding was consistent in direction across the three contributing trials and showed no statistical heterogeneity, although this finding should be interpreted within the context of a limited number of studies. It nonetheless contrasts with the largest randomized trial conducted in this field. The NoPAC trial randomized 496 emergency department patients whose epistaxis persisted after first-aid measures and found no reduction in the need for anterior nasal packing with topical tranexamic acid (43.7% versus 41.3%) and no reduction in recurrent bleeding requiring re-presentation within seven days [20]. That trial included a broader epistaxis population and did not meet the narrowly defined anterior epistaxis population criterion applied in this review. Two further differences are relevant. It used a 200-mg dose delivered by dental roll, whereas the pooled studies used doses ranging from 500 to 1000 mg, and its participants had already undergone a defined period of failed conservative management before randomization. Dose and treatment timing may therefore account for part of the discrepancy. The control preparations in the pooled trials also varied in mechanical and pharmacological activity, including saline-soaked gauze application and vehicles containing topical vasoconstrictors, which may influence the magnitude and interpretation of the pooled estimate. Even so, a neutral result from a trial larger than the entire pooled sample should limit strong conclusions regarding the routine use of topical tranexamic acid as a replacement for anterior nasal packing.

Findings from the present dataset suggest a possible dose-related effect, although this observation is based primarily on a single three-arm trial and requires confirmation. Arikan and Akyol reported absence of bleeding at five minutes in 64.7% of patients receiving 1000 mg, 52.0% receiving 500 mg, and 29.4% receiving saline, with a comparable gradient at 10 minutes. A separate randomized trial in patients taking antiplatelet agents, which included a population underrepresented in the present review, reported faster hemostasis and fewer rebleeding events with topical tranexamic acid than with anterior nasal packing [21]. Dose, timing of application, and baseline hemostatic competence may therefore represent important factors influencing treatment response.

The divergence between the relative and absolute analyses for further hemostatic intervention likely reflects the composite nature of this endpoint rather than an unstable treatment effect. Control-group risk ranged from 28.9% to 64.2% because the three trials defined the outcome differently, including rescue treatment after failure at 15 minutes, salvage tamponade, or any anterior nasal packing during the clinical encounter. These outcomes do not represent identical clinical constructs, and the observed heterogeneity is likely related to variation in outcome definition and baseline risk. With three studies, the Hartung-Knapp adjustment produces wider confidence intervals and reduces the risk of false precision when between-study variance is estimated from a small number of trials [22]. The 24-hour rebleeding estimate should therefore be interpreted with greater confidence than the further-intervention analysis because the outcome definition was more consistent across contributing trials, whereas the effect of topical tranexamic acid on additional hemostatic intervention remains exploratory.

When topical tranexamic acid was compared with anterior nasal packing rather than vehicle-based controls, the effect on early hemostasis was more pronounced. Zahed et al. [14] reported bleeding arrest within 10 minutes in 71.0% of patients receiving tranexamic acid compared with 31.2% receiving packing. Akkan et al. [16] found cessation at 15 minutes in 91.1% of patients receiving atomized tranexamic acid, 93.3% receiving Merocel (polyvinyl acetal sponge) packing, and 71.1% receiving atomized saline, with an advantage of 20.0 percentage points over saline and a nonsignificant difference compared with Merocel. In individual trials, healthcare-utilization outcomes also favored tranexamic acid, including higher rates of discharge within two hours compared with anterior nasal packing and fewer prolonged emergency department stays compared with matched vehicle. These outcomes may be particularly relevant to emergency department workflow and resource utilization.

The trials evaluating hemostatic matrices and packing devices demonstrated a different pattern. Initial hemostasis was statistically indistinguishable between comparators in Singer et al. [11] (90% in both arms), Moumoulidis et al. [13] (81% versus 76%), Murray et al. [15] (76.9% versus 84.6%), and Pouraghaei et al. [17] (79.4% versus 88.5%). However, patient-reported outcomes generally favored the investigational interventions. Floseal (gelatin-thrombin matrix) reduced discomfort, crossover for treatment failure, and otolaryngology consultation compared with conventional packing, whereas oxidized cellulose powder improved satisfaction despite similar bleeding-control times. The main advantage of non-packing strategies may therefore relate more to tolerability and disposition than to clearly superior hemostatic efficacy.

Safety data were limited but reassuring within the available follow-up periods. No serious treatment-related adverse events were reported among trials assessing safety; treatment-related adverse effects did not differ significantly among the 1000-mg, 500-mg, and saline groups, and structured assessment for deep-vein thrombosis, pulmonary embolism, and cerebrovascular events identified no events. However, follow-up in topical tranexamic acid trials did not exceed seven days, limiting assessment of delayed adverse effects. In addition, one trial did not report adverse-event outcomes, and another did not prespecify a safety outcome.

This review has several methodological strengths. Eligibility was restricted to randomized evidence from emergency and otolaryngology settings where acute anterior epistaxis is managed, quantitative synthesis was limited to outcomes with sufficient clinical comparability, absolute effects were reported alongside relative effects, and risk of bias was assessed using the RoB 2 tool [23]. No trial contributing to either meta-analysis was judged at high risk of bias; however, this does not eliminate concerns related to imprecision, indirectness, and clinical heterogeneity. Formal certainty-of-evidence grading was not performed, which limits interpretation of the overall confidence in pooled estimates.

Several limitations qualify these conclusions. Only three trials contributed to each quantitative synthesis, the review was not prospectively registered, and publication bias could not be assessed because fewer than 10 studies contributed to each analysis. The vehicle comparators were not identical and differed in their mechanical and pharmacological properties, while tranexamic acid protocols varied in dose and method of application. Six of the nine trials were open-label, and the limited number of studies precluded subgroup analyses and meta-regression.

Future adequately powered randomized trials should apply standardized definitions of treatment failure, prespecify tranexamic acid dose and delivery method, stratify patients according to anticoagulant and antiplatelet use, and include longer follow-up. Such evidence is needed before the routine first-line role of topical tranexamic acid relative to anterior nasal packing can be clearly established.

## Conclusions

This systematic review and meta-analysis of RCTs suggests that topical tranexamic acid may reduce early rebleeding among adults with acute anterior epistaxis, particularly when compared with matched vehicle controls. However, its effect on reducing further hemostatic intervention remains uncertain due to the limited number of comparable trials, variability in treatment protocols, and differences in outcome definitions. Other nonoperative hemostatic strategies demonstrated potential benefits in selected patient-centered outcomes, although comparative evidence remains heterogeneous. Further adequately powered RCTs using standardized outcome measures and longer follow-up are required to define the optimal role of these interventions in acute epistaxis management.
